# A temporal analysis of the consequences of the drought regime on the water footprint of agriculture in the Guadalupe Valley, Mexico

**DOI:** 10.1038/s41598-024-56407-5

**Published:** 2024-03-13

**Authors:** Vanessa Novoa, Carolina Rojas, Octavio Rojas, Ramón Ahumada-Rudolph, Rebeca Moreno-Santoyo

**Affiliations:** 1 Instituto de Alta Investigación, Universidad de Tarapacá, 18 de Septiembre 2222, Arica, Chile; 2 Facultad de Arquitectura, Diseño y Estudios Urbanos, Instituto de Estudios Urbanos y Territoriales, Instituto Milenio de Socio-Ecología Costera SECOS, Centro de Desarrollo Urbano Sustentable CEDEUS, Pontificia Universidad Católica de Chile, El Comendador 1916, Providencia, Santiago, Chile; 3 Departamento de Planificación Territorial y Sistemas Urbanos, Facultad de Ciencias Ambientales, Centro EULA, Universidad de Concepción, Víctor Lamas 1290, PO Box 160-C., Concepción, Chile; 4 Laboratorio de Química Aplicada y Sustentable (LabQAS), Departamento de Química, Facultad de Ciencias, Universidad del Bío-Bío, Avenida Collao 1202, PO Box 5-C., 4051381 Concepción, Chile; 5 Facultad de Ciencias Marinas, Instituto de Investigaciones Oceanológicas, Universidad Autónoma de Baja California, Carretera Ensenada-Tijuana 3917, Ensenada, Baja California Mexico

**Keywords:** Drylands, Arid, Semi-arid, Blue- Green- Grey water, Water scarcity, Environmental sciences, Hydrology, Hydrology

## Abstract

Changes in water availability have a substantial impact on the sustainability and maintenance of agriculture, with water footprint (WF) being a robust methodology to assess these transformations. The Guadalupe Valley is one of the places with the highest agricultural production in Mexico. Despite its semi-arid climatic conditions, it provides high-quality crops that are well-positioned in the world. The historical trend of rainfall and temperatures between 1987 and 2017 was analyzed to identify climatic patterns in the territory. Through the calculations of the water footprint of Grapevine and Olive crops, the sensitivity of the crops to recurrent water deficit and their adaptation in their yields to drought episodes was identified. The reduction in precipitation and occurrence of extreme temperatures have contributed significantly towards augmenting crop evapotranspiration and, consequently, intensifying crop irrigation demands. As a result, there has been an apparent increase in the consumption of WF_agricultural_ since 2007. Thus, the period of highest WF_agricultural_ consumption was 2014 (Extremely dry), as opposed to 2011 (Very wet). In particular, the lowest WF_green_ consumptions were observed in extremely dry years, that is, > 20% of the WF_agricultural_ intensifying drought events. Therefore, these periods were compensated with higher uses of WF_blue_ and WF_gray_, which are inversely correlated with precipitation, where vine crops consume 73% more WF_agricultural_ compared to olive plantations, showing greater interannual variability. These results contribute to analyzing the temporal evolution of water consumption for agriculture, providing a basis for rational water use strategies.

## Introduction

At present, droughts are one of the extreme socio-natural hazards that pose the greatest severity or threat to social stability and biophysical resilience of the environment, due to their repercussions on agricultural production and economic growth^1^. Moreover, drought processes develop progressively within a given territory due to a continuous and cyclical period of water stress, resulting from the abnormal decrease in precipitation, together with fluctuating temperatures and eco-hydrological changes^2^.

It is essential to consider that the water cycle in environments affected by drought is characterized by high evapotranspiration rates and reduced precipitation, resulting in deficient surface runoff and low groundwater recharge. The latter leads to decreased soil moisture storage, increased erosion, and deteriorated vegetation cover in areas far from river sources^3–5^.

In addition, droughts can be categorized into meteorological, agricultural, hydrological, and socioeconomic types based on their periodicity. They often lead to water shortages or deficits, particularly severe in arid or semi-arid regions^6^. These regions constitute 41% of the global area and are experiencing trends in water availability that fall below historical averages^7^. It is projected that dryland extent will increase by 4% to 10% by 2100 compared to the 1961–1990 period under the RCP4.5 and RCP8.5 climate change^8,9^.

Water scarcity is a significant issue in various parts of the world. The critical areas affected by it include the western USA, Mexico, the west coast of South America, sub-Saharan Africa, Pakistan, the Middle East, and Central America^5,8^. Nevertheless, the highest percentage of drylands is concentrated in Latin American and Caribbean countries, that is, Argentina (69%), Mexico (65%), and Chile (58%)^10^.

Furthermore, the combination of climate change, climate variability, and inefficient water usage^11^, along with irrational consumption habits^12^, intensified agriculture, and changes in land use^13^, have significantly impacted the load capacity of water resources and their security. This has led to a severe water crisis in arid areas^14^. As a result, conflicts related to socio-environmental issues have become more severe in various regions, hindering the achievement of the United Nations' Sustainable Development Goals (SDGs). Specifically, it is making it difficult to achieve the targets of Zero Hunger (Target 2), Clean Water and Sanitation (Target 6), and Life on Land (Target 15) by 2030^15,16^.

However, it is not only about the amount of water that is limited by climatic variables or overexploitation of water sources, but also about the repercussions in geographical and temporal dimensions that influence the hydro-social cycle^17^. As agriculture is one of the main productive activities globally, extreme droughts have had an impact on the loss of agricultural land, estimated at approximately 21 t^−1^ ha^−1^ average, which is up to 16 times higher than the weathering process of rocks^14^.

Understanding freshwater consumption in agriculture is crucial^18^ to establishing resilience and food security strategies. Therefore, broadening the concept towards an environmental water flow approach becomes necessary. This approach will help in identifying water-saving routes in regional crop production. The assessment of water resources appropriation needs to broaden the criteria for integrated resource management. The focus should be on determining the various environmental sources or flows of water for effective and sustainable management of water resources^19^.

It is essential to consider not only the use of blue water (water resources extracted from surface water runoff and groundwater infiltration) but also the use of green water (precipitation temporarily stored in the soil or intercepted by vegetation and exported to the atmosphere as evapotranspiration), along with their interactions^20^. The distribution of seasonal precipitation, combined with evapotranspiration, directly influences water availability patterns, affecting regional hydrology and crop yields^21^.

Consequently, the main functions of green water are productive (i.e., maintenance of up to ~ 65% of overall crop production) and regulatory (i.e., energy balance and convective conditions)^22^, where green water flow is composed of about 59% transpiration, 21% plant interception, 10% soil interception, and 6% soil moisture evaporation^23^.

Similarly, blue water serves a multitude of functions, including but not limited to supply, transport/chemical loading (nutrients and pollutants), regulation/control, and production^24,25^; furthermore, irrigation accounts for about 70% of the world's blue water use^26^.

A complex interaction of these variables (climatic and productive) has led water-scarce regions to produce more water-intensive but higher-value crops. In regions where water resources for agricultural purposes are scarce, a potential reduction of 56% in water usage can be achieved by cultivating crops better adapted to the area's green and blue water availability^27^.

At the same time, it is essential to acknowledge that soils in arid regions are fragile (eroded or degraded). This is because they have less natural primary production, organic matter, nutrients, water retention capacity, and fertility. As a result, plant growth and biomass are negatively affected. Therefore, before adding excessive amounts of chemical fertilizers, it is essential to assess the volume of grey water needed to assimilate the pollutants generated during the cultivation process^28^.

Considering the previously mentioned, prioritizing the evaluation of water abstractions is crucial for understanding the relationship between productive activities, the water cycle, and the pressure on water resources to ensure sustainable development in agriculture. Comprehensive indicators such as estimating the water footprint can help achieve this goal^20^.

In addition, the agricultural water footprint (WF_agriculture_) is a measure that indirectly evaluates the impact of aridity by considering the local water demands and supplies^23^. It considers atmospheric water requirements such as evaporation and evapotranspiration, as well as temperature, precipitation, and irrigation. The measure also considers soil moisture, soil characteristics, and crop yield^21,29^.

WF_agriculture_ studies distinguish between the use of different water sources—blue (WF_blue_), green (WF_green_), and gray (WF_gray_)—where the amount of precipitation before the growing season can reduce the need for irrigation during crop growth. This can lead to a significant decrease in the use of blue water up to 96% for some crops^8,20,30^.

However, during drought events, scarcity affects water flows equally, becoming crucial to increase irrigation to compensate for crops' deficit or green water requirements (CWU_green_). Nevertheless, more than blue water requirements (CWU_blue_) are required, constituting deficit irrigation at the expense of environmental flows and/or groundwater reserves^30–32^.

Thus, to contribute to land management programs for irrigated crops in water-stressed basins, it is crucial to have accurate information on how agricultural water footprints have changed over time. This requires refined estimates over extended periods, which can be used in decision-making to reduce the latency of input data and improve future projections. By incorporating the influence of intra- and inter-annual climate variability in drought mitigation or action plans, we can make more informed decisions based on water footprint results^33^.

Our research lies in understanding water use in agriculture, considering that in Mexico, approximately two-thirds of its territory is located in arid or semi-arid areas facing natural water scarcity, where the pressure on water resources has experienced a severe drought condition, which has persisted in the last 27 years, associated with El Nino Southern Oscillation (ENSO) and by the increase in water demand (up to 35%), especially after the mid-1990s^34–36^.

The water deficit is particularly severe in northern Mexico, where semi-arid areas cover about 50% of the total surface^37^. The central forecasts correspond to a 10–20% decrease in mean annual precipitation, a 1.5 to 2.5 °C temperature increase, intensified incidence of heat waves, and a reduction in aquifer recharge^38^.

The Valle de Guadalupe is a semi-arid rural area in the Ensenada municipality in Baja California. Despite the critical water situation, it is one of Mexico's highest producers of olives and grapevines, with about 46.6% of the total cultivated area dedicated to high-quality, high-value grapes^39^. The area produces 75% of Mexico's wine and 30% of its olive oil, making export crops and by-products the primary economic and tourism livelihood source. However, the limited water resources and agricultural land make the continuity of these industries vulnerable.

Different crops require varying amounts of water, and it is crucial to assess their water footprint to understand the factors that influence these differences. This helps promote the efficient use of water in agricultural production. To this end, this study examines the water footprint for olive and vine cultivation, two of the most predominant crops. Additionally, the impact of climate variability on agricultural WF_agricultural_ consumption is analyzed through temporal analysis. This analysis is based on the climatic variables recorded for very wet, wet, normal, dry, and arid years.

## Results

### Determination of climatic variability

**Figure 1:**
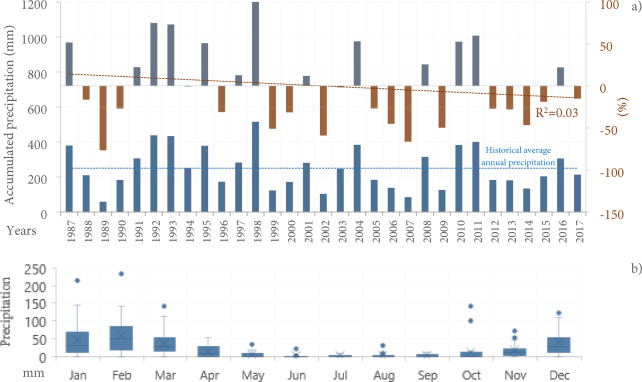
Precipitation behavior from 1987—2017 of the Guadalupe Valley. (**a**) Percentage anomaly of total annual precipitation: blue bars represent positive values (surplus), and red bars represent negative values (deficit) and accumulated frequency of annual precipitation in both climatological stations Agua Caliente and El Porvenir, (**b**) Trends of monthly periods (boxplots): the horizontal line and square inside the box indicate the median and mean. The lower and upper ends of the box correspond to the quantiles 0.25 and 0.75. Lower and upper whiskers: quantile 0.05 and 0.95, respectively.

The interannual precipitation of the Guadalupe Valley for the 29 years analyzed shows a historical mean of 250 mm, with 62% of the analyzed years below this mean. The years 1989, 1999, 2002, 2007, 2009, and 2014 recorded amounts between 59–133 mm, which is significantly lower precipitation compared to the other years (F_(30,341)_ = 1.03; p = 0.001) (Fig. 1a). That is, years with a deficit between 76 to 47% of precipitation regarding the 1987–2017 average, with a negative trend starting in 2005 (Fig. 1a).

In the annual cycle, the behavior of seasonal cycles and extreme values in daily and monthly observations indicate greater variability and rainfall amounts during the winter period (December-March), where about 74% of precipitation occurs (ANOVA F_(11,360)_ = 16.8, p = 0.001), with a prolonged dry period in June–August (Fig. 1b).

**Figure 2:**
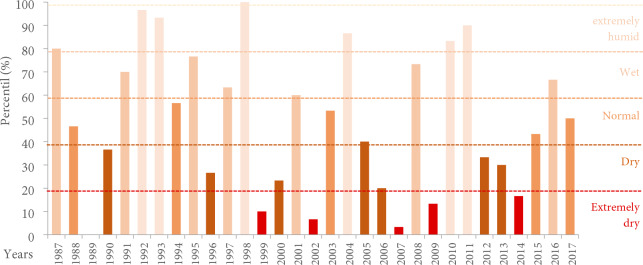
Climate classification according to the precipitation thresholds in the Guadalupe Valley.

According to the percentile analysis, extreme conditions of very dry years were observed in 1989, 1999, 2002, 2007, 2009, and 2014 with 0—20% of the precipitation percentile, with 1989 being the most extreme. Contrary to the years 1987, 1992, 1993, 1998, 1998, 2004, 2010, and 2011 which showed a very wet condition, between 80–100%. Only five years of the analyzed series were classified as normal (Fig. 2).

**Figure 3:**
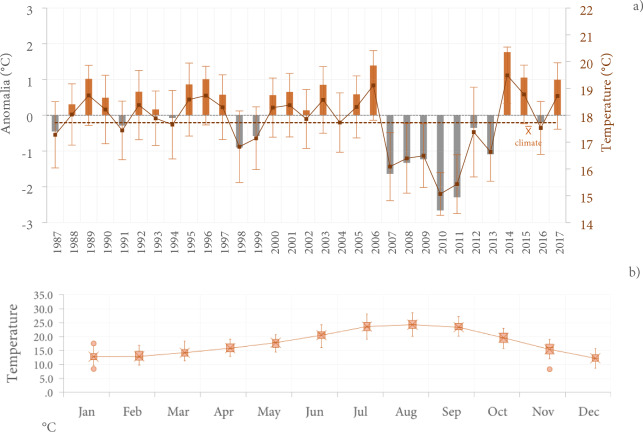
Evolution of the average temperature of the Guadalupe Valley. (**a**) Absolute anomalies of the average annual temperature for the 1987–2017 period (red bars indicate warming, and blue bars indicate cooling) and annual average, (**b**) monthly average.

Regarding temperature, annual mean values ranged from 15° to 19.5 °C, with the years 2006 and 2014 being the warmest with recorded temperatures above 19 °C, that is, positive anomalies of + 1.4 °C to + 1.8 °C above the climatological average in 1987–2017 series (30 years), where it is possible to observe a dominance of the natural climate variability signal, as opposed to ENSO years (contributing to warmer temperatures, overlapping with the El Niño period 2005–2006 or 2014–2015) (Fig. 3a).

The evolution of the monthly mean temperature shows significantly higher values in the summer period (July, August, September) compared to other months of the year (ANOVA F_(11,360)_ = 170.2, P = 0.001) (Fig. 3b).

Lastly, the precipitation and temperature data series are moderately inversely correlated (r = − 0.66; p < 0.05), i.e., precipitation is lower in the months where temperature increases, decreasing water availability in the Guadalupe Valley in the warmer periods.

In summary, high climatic variability of the Guadalupe Valley can be observed from 1996, increasing both ‘very dry’ and ‘very wet’ years, thus intensifying extreme weather conditions. As a result, the occurrence of extreme meteorological droughts has been evident every 3 to 5 years since 1999.

The annual precipitation trend has decreased over the last 12 years; however, 1989 stands out for its low rainfall and high temperatures. Meanwhile, the changes identified in the annual temperature result in an amplification of the thermal oscillation.

### Water footprint of the Guadalupe Valley for the agricultural sector

**Figure 4:**
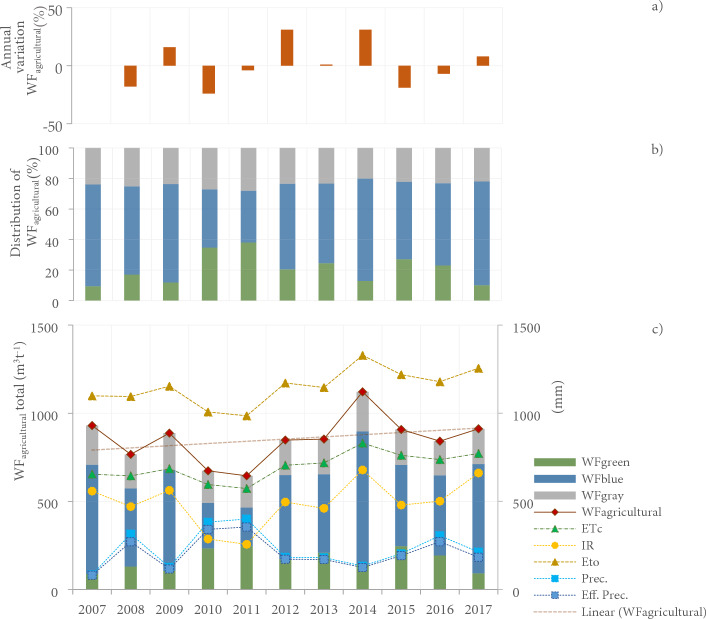
(**a**) Annual variation percentage of WFagricultural regarding the previous year. (**b**) Annual distribution in WFblue, WFgreen, and WFgray consumptions. c) Accumulated WFagricultural of the Guadalupe Valley basin, Mexico, crop evapotranspiration (ETc), irrigation requirements (IR), reference evapotranspiration (ETo), annual precipitation (Prec.) and effective precipitation (Eff. Prec.) from 2007–2017.

Crop evapotranspiration (ETc) in 2007–2017 was estimated between 573 and 830 mm. It is observed that ETc values decreased in 2010–2011, i.e., 596—573 mm respectively, unlike 2014, where it reached the maximum amounts. The temporal behavior of ETc is correlated with ETo (r = 0.99; p < 0.05) and irrigation requirements (IR) (R = 0.93; p < 0.05) (Fig. 4).

Similarly, the IR of the crops is inversely correlated with precipitation and effective precipitation (r = − 0.84; p < 0.05), which were higher in 2010–2011. With the increase in precipitation, ETo and ETc values were drastically reduced, combined with the decrease in temperatures, as in the case of 2010–2011. In contrast, in 2014, although it was not the only extremely dry year with a precipitation deficit, the extreme temperatures recorded led to an increase in ETc and IR.

The WF_agricultural_ of the crops analyzed in the Guadalupe Valley presented estimated consumptions between 645 and 1121 m^3^/ton. Although there was some interannual variability, a slight incremental trend was identified in the 11 years analyzed, and the year 2014, which had extremely dry rainfall, had the highest WF_agricultural_ consumption. On the other hand, 2011, which was a very wet year, had the lowest consumption estimated (as shown in Fig. 4).

Thus, WF_agricultural_ is significantly correlated with ETo (r = 0.86; p < 0.05), ETc (r = 0.88; p < 0.05), IR (r = 0.90; p < 0.05), WF_blue_ (r = 0.95; p < 0.05), and WF_gray_ (r = 0.93; p < 0.05), and inversely correlated with precipitation (r = − 0.85; p < 0.05) (Fig. 4).

The highest consumption of WF_green_ was observed in 2010, 2011, and 2015 (Extremely humid and normal), with 35%, 38%, and 27% of the total WF_agricultural_, being found in these periods the precipitation and soil moisture necessary for crop growth, a variable that is inversely correlated with IR (r = − 0.78; p < 0.05).

While the highest uses of WF_blue_ were calculated for Extremely dry years, when the need for irrigation increased, that is, the years 2007 (620 m^3^t^−1^), 2009 (571 m^3^t^−1^), 2014 (752 m^3^t^−1^) and 2017 (620 m^3^t^−1^), volumes corresponding to 64—68% of the WF_agricultural_. Additionally, WF_blue_ trends are correlated with ETo (r = 0.81; p < 0.05), ETc (r = 0.80; p < 0.05), IR (0.97; p < 0.05), and WF_gray_ (0.95; p < 0.05) and inversely correlated with precipitation (r = -0.87; p < 0.05).

Meanwhile, the highest uses of WF_gray_ were determined in 2007, 2009, 2014, and 2015 (Extremely dry and normal) with consumption above 200 m^3^t^−1^, a variable correlated with irrigation requirements (r = 0.82; p < 0.05), and inversely correlated with precipitation (r = − 0.91; p < 0.05) (Fig. 4).

### Water footprint by crop type

**Figure 5:**
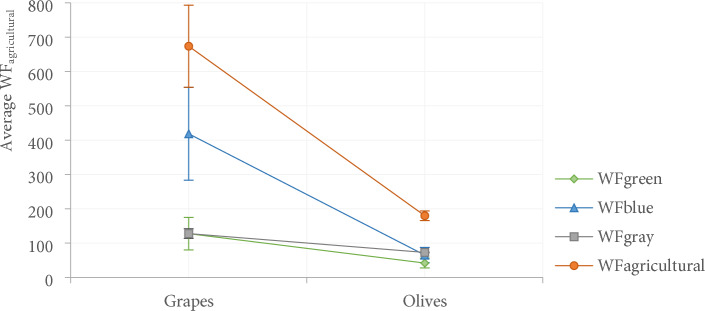
Average of WF_agricultural_ (WF_green_, _blue_, _gray_) the grapes and olives crops, series analyzed for the Valle de Guadalupe, Mexico.

Significant differences were observed in the WF_agricultural_ consumption of the evaluated crops, with higher consumption in grapevine plantations by 73%, corresponding to an average of 127.4 ± 11.8 m^3^t^−1^ between 2007 – 2017 (F_(1,20)_ = 185.1; p = 0.001), as well as the consumptions of WF_green_, WF_blue_, and WF_gray_, which are significantly higher in grape production compared to olive, that is, 67%, 84%, and 43%, respectively (F_(3,30)_ = 129.7; p = 0.001) (Fig. 5).

**Figure 6:**
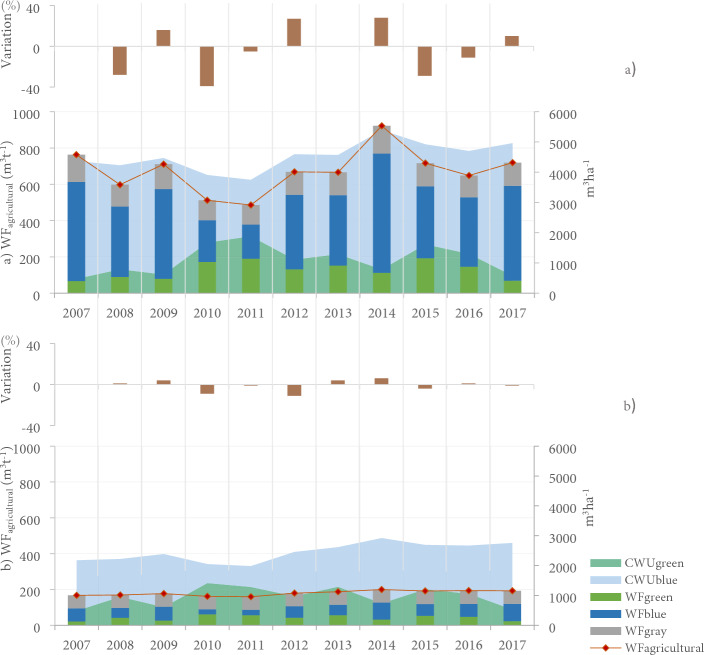
Annual variation of WF_agricultural_ consumption of crops: a) grapes b) olive analyzed in 2007–2017 for the Valle de Guadalupe, Mexico.

Grapevine crops show high interannual variability, that is, an average of 3% ± 22.5 in WF_agricultural_ consumption, with a maximum of 922.1 m^3^t^−1^ in 2014 (Extremely dry), presenting a higher use of WF_green_ in 2010, 2011, and 2015, which is above the 170 m^3^t^−1^ uses of WF_blue_ in 2007, 2014, and 2017, exceeding 500 m^3^t^−1^, and WF_gray_ in 2007–2014, exceeding 150 m^3^t^−1^. Maximum water uses of CWU_green_ crops were evinced in the 2011 period (Extremely humid) (1883 m^3^ha^−1^) and for CWU_blue_ in 2014 (Extremely dry) (4601 m^3^ha^−1^) (Fig. 6a).

On the other hand, olive crops presented a lower interannual variability of WF_agricultural_, that is -1% ± 5.3, with amounts up to 199 m^3^t^−1^ recorded in 2014 (Extremely dry). The highest consumptions of WF_green_ were in 2010, 2011, and 2013, above 55 m^3^t^−1^; WF_blue_ in 2014–2017 with values above 95 m^3^t^−1^; and WF_gray_ in 2016 with more than 74 m^3^t^−1^. In addition, in the 2010 period, the maximum uses of CWU_green_ (1414 m^3^ha^−1^) and 2014–2017 of CWU_blue_ (> 2000 m^3^ha^−1^) were estimated (Fig. 6b).

Crop conditions in the Guadalupe Valley reported yields below 10 t^−1^ ha^−1^ for grapevine^39,40^and 12 t^−1^ ha^−1^for olive^41^. Therefore, the net crop production and annual WF_agriculture_ consumptions, when compared to the amount of water granted (25 × 10^6^ m^3^ according to the Drinking Water Committee), it is possible to identify the annual use of 36.4 to 82.7% of the available water (Table 1), where the maximum Total WF_grapes_ were evinced in 2012, 2013, 2014 and for Total WF_olives_ in 2010, 2013, 2016 (Table 1). Both crops accounted for an average of 55% ± 12 of the water available for agriculture in the study period.

**Table 1:** WF_grapes_ and WF_olives_ consumption according to annual production and water availability in the Guadalupe Valley, Mexico. Source: SIAP, IMIP.

Years	Grapes production (t)	Total WF_grapes_ (m^3^)	Olive production (t)	Total WF_olives_ (m^3^)	Total WF_grapes +_ WF_olives_ (m^3^)	Fraction used (%)
2007	16,264.5	12,413,936.5	1968.6	329,074.6	12,743,011.1	51.0
2008	14,658.8	8,761,143.6	2009.3	339,631.3	9,100,774.8	36.4
2009	19,099.9	13,574,360.8	1814.0	320,602.7	13,894,963.5	55.6
2010	22,514.3	11,524,383.7	7196.8	1,162,996.2	12,687,379.8	50.7
2011	21,820.4	10,602,768.1	260.0	41,424.1	10,644,192.1	42.6
2012	23,146.0	15,470,895.8	1985.1	355,914.4	15,826,810.2	63.3
2013	22,349.4	14,890,058.8	5811.0	1,087,748.4	15,977,807.2	63.9
2014	22,411.2	20,664,300.5	0.0	0.0	20,664,300.5	82.7
2015	19,357.2	13,868,140.3	160.4	30,711.8	13,898,852.1	55.6
2016	15,919.6	10,319,803.6	4851.3	938,202.3	11,258,005.9	45.0
2017	20,678.5	14,875,620.4	2329.5	448,248.6	15,323,869.1	61.3

## Discussion

Currently, drought management comprises measures aimed at restricting water supply, reducing demand, and minimizing impacts. These measures have multiple effects on the maintenance and conservation of ecosystems, optimization of water supply, availability, and recharge within watersheds^4^.

In order to use resources efficiently, it is important to understand the consequences of climate variability on hydrological responses. In terms of precipitation in the Guadalupe Valley, in the last 30 years analyzed, 62% of the period is below the historical average of 250 mm, i.e., a deficit of 76—47% of precipitation, with a clear negative annual/seasonal trend since 2005.

The yearly variation in precipitation levels can be attributed to the impact of El Niño-Southern Oscillation (ENSO) and Pacific Warm Decadal Oscillation (PDO) events^42^. Our analysis has identified extreme conditions such as very dry or wet years and their alternation as evidence of these events.

Therefore, it is important to note that the transition from El Niño during 1998 (the rainiest year in the hydro-climatic record, classified as very wet) to the La Niña event (the following year) (Fig. 1), which marked the beginning of prolonged drought periods, in which extreme drought became even more severe later, that is, in the years 1999, 2002, 2006, 2006, 2007, 2009, and 2014 (P < 140 mm), being possible to observe a dominance of the natural climate variability signal, versus ENSO years, contributing to warmer temperatures as El Niño in the 2005–2006 or 2014–2015 periods observed, with positive anomalies from + 1.4 °C to + 1.8 °C.

El Niño is linked to the increase in surface water temperature in the central Pacific Ocean, inducing severe droughts in Indonesia, Australia, and northeastern South America^5,43^. La Niña, on the other hand, occurs when the surface water temperature drops off the coast in the Pacific Ocean, contributing to drier than normal weather in the North and South American regions, which also influences drought episodes, with a more complex impact on weather patterns than El Niño^5,43,44^.

Although El Niño-Southern Oscillation (ENSO), Pacific Decadal Oscillation (PDO) and Sea Surface Temperatures (SST) of the tropical Atlantic have played an important role in recent drought in northern Mexico^44,45^. However, global warming has increased the demand for atmospheric humidity and has probably altered circulation patterns, contributing to sustained episodes of drought in the northwestern region of Mexico, observed since 1994^34^, where recurrent dry years have significantly diminished water resources, in which high interannual climate variability through changes in precipitation and temperature, aggravating the present situation in the Guadalupe Valley^10^.

Temperature and precipitation are the main climatic factors that produce changes in soil water content and, therefore, directly regulate crop growth. By including these variables, the water footprint analysis reflects the water requirements in agriculture and the sensitivity of the results, where the precipitation deficit and extreme increase in temperatures conditioned the increase in ETo, ETc and, IR^46^.

Therefore, of the 11 years evaluated and considered as the present conditions (2007–2017), a slight incremental trend of water consumption was observed, despite the interannual variability, where the highest consumption of WF_agricultural_ (grapevine + olive) was in the year 2014, classified as Extremely dry. This trend was also identified in the high WF_blue_ consumption in 2007, 2009, 2014, and 2017, since during drought years, there was an increase in crop ET related to climatic variables and irrigation requirements.

**Figure 7:**
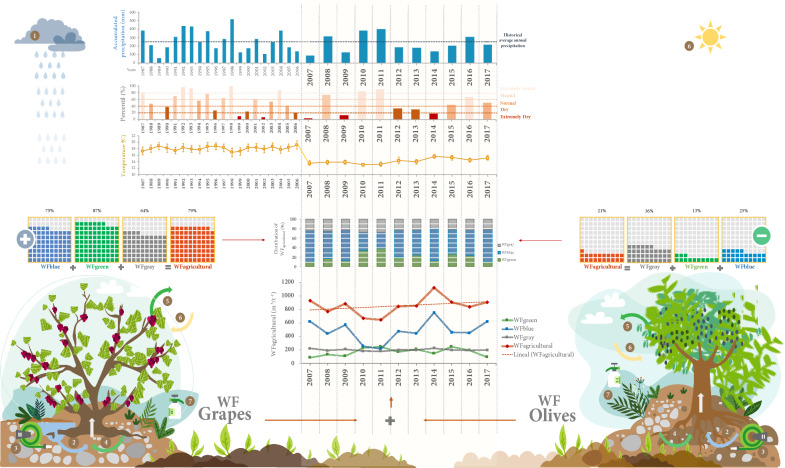
Summary of the agricultural water footprint of the Guadalupe Valley (Reference: Author’s elaboration).

In contrast, in the years classified as extremely wet and normal, i.e., 2010, 2011, and 2015, more WF_green_ was consumed; being essential to include the evapotranspiration flux link between the ecological and hydrological systems, understanding that biological water use is inexorably linked to ecosystem productivity (Fig. 7).

The reported results, as described in Marston and Konar^25^ in the California Valley, differences in WF_agricultural_ during periods of drought are also attributed to reasons for changes in crop area and yields, increases in ETc, irrigation demand to compensate for precipitation deficits, and increased groundwater consumption due to reduced surface water availability^47^.

In these wine and fruit growing regions, in addition to the seasonal climatic changes described above, it is also necessary to include the influence of climate on an intermediate geographical scale (< 50 km) since these local climatic differences can cause considerable changes in crop physiology and phenology. Therefore, it is suggested to grow deep-rooted plants in drylands, due to the lower probability of suffering stress due to effects or changes in soil moisture, compared to shallow-rooted crops with less root coverage^48,49^.

However, crops can also modify their water needs through physiological adjustments, that is, in the regulation of stomatal conductance and leaf area transformation^50^, being crucial to the adaptation of plants to changing hydrological regimes, where their growth depends on whether the altered water supply is sufficient. Thus, the highest water requirements per hectare of crops were recorded in 2011 (1883 m^3^ha^−1^) for CWU_green_ and 2014 (4601 m^3^ha^−1^) for CWU_blue_, with olive crops being the best adapted to interannual variability according to WF_agricultural_.

As WF_agricultural_ is associated with both yield and seasonal water consumption, grapevine growing conditions in the Guadalupe Valley are limited by low yields, i.e., less than 10 t^-1^ ha^−1^, compared to the main wine producers, for example, Italy, France, and Spain (over 80 t^-1^ ha^−1^). For olives, yields are even more extreme, reaching maximums of 23.40 t^−1^ ha^−1^ and minimums of 0.10 t^−1^ ha^−1^, respectively^35,37^.

Excessive use of nitrogen fertilizers is common in sandy soils such as those in the Guadalupe Valley. As a result, the soil contains nitrate levels of more than 15 mg kg^−1^, while nearby aquifers show levels of 10 mg L^−1^^51^. This has led to severe contamination problems by leaching and accumulation of macronutrients, especially during extremely dry periods when more irrigation is needed. As a solution, significant uses of WF_gray_ were established to prevent further contamination^52^.

For agriculture to be a sustainable activity over time, it is necessary to include all approaches for mitigation and adaptation to water-scarce environments, from broadly tolerant crops to the introduction of climate and disease-resistant seed varieties, diversification, and crop calendars. In addition to the above, better information on droughts and climate variables, planned and comprehensive agricultural relocation, support for land use changes, and marketing strategies^53^ become necessary, with productive land and fertile soil being the most important natural capital asset^27^. Therefore, soil preparation is essential, such as zero tillage and composting, the introduction of cover crops, intensive grazing management, and reduction in the use of chemical and synthetic inputs. Banwell^54^ insist that such measures should consider the participation of various stakeholders, especially with a bottom-up approach.

Indeed, only by modifying the cropping pattern could water scarcity be significantly reduced and production increased. According to Davis^56^, it is possible to reduce green and blue water consumption by 13.6% and 12.1%, respectively, only by growing the crops recommended for the area. First, however, farmers and decision-makers must be aware of the problem.

Since drought or water scarcity are dynamic, multiple indicators for their monitoring have been established^57^. Although initially, the water footprint is a tool for the evaluation of water consumption in production chains, in this study, it is possible to establish the sensitivity of crop water consumption results to changing weather conditions^58–60^, contributing to be the starting point for timely monitoring of water deficit and agricultural development in the area, for adequate preparation of responses according to the effects on the environment, agriculture, and local economy, understanding that the beginning and end of dry periods are perceived in retrospect. However, food security and water availability need immediate action^48,61^.

Lastly, we must not forget that the water resource is a priority requirement on which we depend, fulfilling its function through its cycle, storage, transport, hydroclimatic and ecological regulation, in which water flows, in all its states (blue, green, and gray), intervene in energy balances and biogeochemical cycles, for example, critical ecosystem services require 90% of global evapotranspiration for their functioning^62,63^.

## Conclusion

The increase in precipitation in the Guadalupe Valley has a significant impact on the drastic reduction in ETo and ETc values, also influenced by the decrease in temperatures. However, this situation was contrary to the precipitation deficit and extreme temperatures that caused an increase in ETc and IR in crops.

A clear incremental trend in the consumption of WF_agricultural_ was identified during the 11 years analyzed. Moreover, 2014 (Extremely dry) was the period with the highest WF_agricultural_ consumption, as opposed to 2011 (very wet), where the lowest consumption was estimated.

The quantification of green water flow must necessarily be included in the evaluation of water resource security since the lowest consumption of WF_green_ was observed in extremely dry years, that is, > 20% of the WF_agricultural_, due to the limitation in the availability of green water, intensifying and aggravating drought events. Periods compensated with higher uses of WF_blue_, corresponding to 64—68% of the WF_agricultural_, periods where the need for irrigation increases.

The dissolution of fertilizers (nitrogen) decreases in periods of water scarcity, thus increasing the probability of contamination of environmental matrices, where the highest WF_gray_ was determined in Extremely dry years with a consumption of over 200 m^3^/ton, a variable correlated with the irrigation requirement and inversely correlated with precipitation.

Grapevine crops consume 73% more WF_agricultural_ compared to olive plantations and also showed greater inter-annual variability, in other words, less adaptation to climate variability. Nevertheless, the commercialization and water productivity of these crops is responsible for the continuity of their production.

The results obtained are a significant step towards reducing water waste in agriculture in arid regions with limited resources and fragile ecological environments. Despite the efforts made so far, such as the National Program Against Drought, the Mexican Drought Monitor, and the Programs for Drought Prevention and Mitigation Measures, these measures are considered insufficient. We must formulate strategies involving the rational use of agricultural water to tackle this issue effectively.

## Materials and methods

### Site of study

The Guadalupe Valley basin (32°03’N—116°37’W) is located northeast of the municipality of Ensenada, Baja California, Mexico (2380 km^2^). Its population reaches 6,924 inhabitants, concentrated in 0.4% of the basin’s territory, 38.7% of the inhabitants live in poverty, and 35.5% are vulnerable due to social deprivation. Economic activities are mainly related to livestock, tourism, and agriculture^64^.

**Figure 8:**
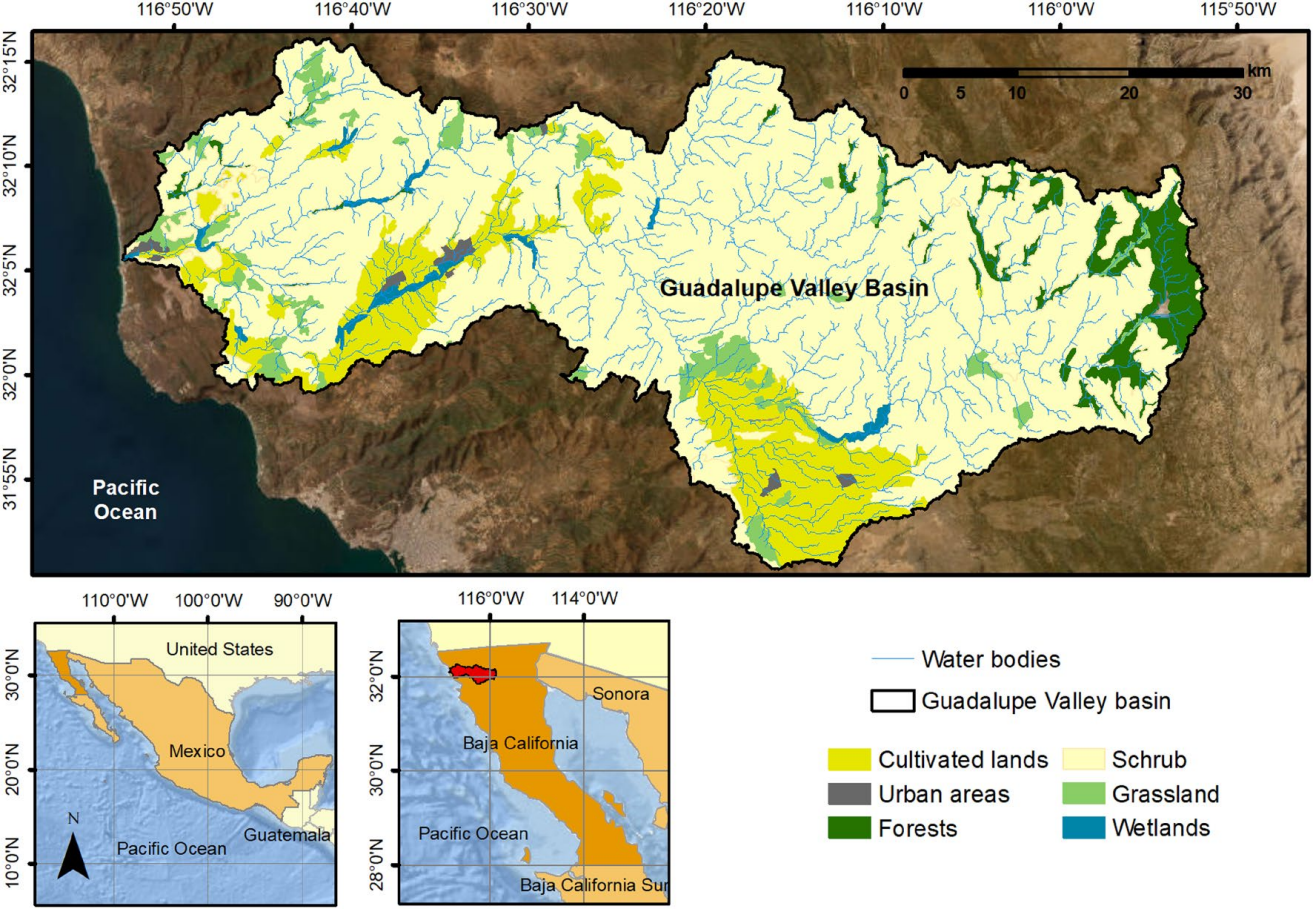
Location, land use, and water bodies identified in the study area, Guadalupe Valley, Mexico (Reference: ArcGIS 9.3 software developed by ESRI (Environmental Systems Research Institute), https://www.esri.com)^66^.

Similarly, the little of the land in this area is used for agriculture (cultivated lands), accounting for 2.7% of the total land area (6272 hectares). Out of this, 60% is dedicated to grapevine and olive production. Other land uses include scrub (94.3%), forest (1.8%), grassland (1%) and water bodies (0.02%)^64,65^ as seen in Fig. 8.

The Guadalupe Valley is classified as a semi-desert region with a semi-arid Mediterranean climate, directly influenced by the Pacific Ocean coastal system, which is only 25 km away, exposing Guadalupe Valley to dense fogs, breezes, and marine humidity. Climatic conditions resulted in mild temperatures year-round, with an average of 18 °C annually from 1986–2016. Monthly temperatures range from 5.4 to 31.6°C^67^.

Precipitation is a consequence of the weakening of the North Pacific system in the winter, which allows the entry of convective and frontal systems; average annual rainfall amounts reach 298 mm (1992—2016)^68^. Precipitation variations are mainly associated with El Niño/Southern Oscillation and the Pacific Decadal Oscillation (PDO). These hydroclimatic phenomena bring unusual precipitation events to the region, as under warm conditions during El Niño and the PDO, the precipitation rate tends to increase. In contrast, under cold conditions during El Niño and the PDO, the precipitation decreases^45,69^. The research methodology was developed in 3 stages. In the first stage, the climatic behavior of the Guadalupe Valley basin was analyzed. During the second stage, the database was developed, and the classification of the drought cycles was conducted. Finally, the crops’ water footprint was calculated.

### Determination of climatic variability

The meteorological input data were selected from 2 meteorological and pluviometric stations maintained by the Information System for Irrigation Water Management of Baja California (Fomento Agropecuario de Baja California) and the meteorological network CICESE-CLICOM (Center for Scientific Research and Higher Education at Ensenada). These stations are located in the Guadalupe Valley, that is Agua Caliente (32°04'N–116°37'W) and Porvenir (32°06'N–116°27'W) stations, considering a 31-year data (1987–2017 period). Monthly, seasonal, and annual precipitation and temperature data were used.

Exploratory and consistency analyses of the data were carried out using the double-mass curve method^70^. Boxplots were used to visualize the behavior, which provided information on the trend of seasonal cycles and extreme values in the observations. The classification of dry, normal, and wet years was made using the percentile method, which defines five categories: very dry (0–20 percentile), dry (20–40 percentile), normal (40–60 percentile), wet (60–80) and very wet (80–100)^71^.

### Calculation of the water footprint of olive and grapevine crops (WF_agricultural_)

In the WF_agricultural_ analysis, climate variables were included with georeferenced information from 2007—2017, with primary values of precipitation (mm/month), temperature (°C), wind (km/day), insolation (hrs), humidity (%), and secondary values of effective precipitation (mm/month), evapotranspiration (mm/day), solar radiation (MJ/m^2^/day), as well as standardized soil properties data.

In addition, grapevine and olive crop data were used, such as Kc values, stages, and critical depletion fraction by development period, along with estimates of crop evapotranspiration and irrigation requirements.

To estimate the WF_agricultural_, the methodology proposed by Hoekstra^72^ was used, which considers the sum of its components: WF_blue_, WF_green_, and WF_gray_, of the assessed crops grapes (WF_grapes_) and olives (WF_olives_) (Eqs. (1) and (2).

The determination of WF_blue_ and WF_green_ was made from Eqs. (3) and (4), where Y corresponds to crop yield (t ha^-1^) and CWU_blue_, CWU_green_ green and blue crop water use, according to its source (precipitation or irrigation), expressed in m^3^ ha^−1^.

For the calculation of blue and green crop water use (CWU_blue_, CWU_green_), they were estimated based on the relationships of Eqs. (5) and (6). In the equations, Σ represents the crop growth cycle, that is, which starts from sowing (day 1) and ends at harvest. In addition, Igp represents the length in days of each cycle stage. ET_blue_ is blue water evapotranspiration, and ET_green_ is green water evapotranspiration. We use a conversion factor 10 to convert the water depths in millimeters into water volumes per land surface area in m^3^/ha.

1 $${\text{WF}}_{{{\text{agricultural}}}} = \, \Sigma {\text{WF}}_{{{\text{crops}}}} \left( {{\text{WF}}_{{{\text{grapes}}}} + {\text{ WF}}_{{{\text{olives}}}} } \right) \,{\text{m}}^{{3}} {\text{t}}^{{ - {1}}} \,$$

2 $${\text{WF}}_{{{\text{crops}}}} = \, \Sigma {\text{WF}}_{{{\text{green}}}} + {\text{ WF}}_{{{\text{blue}}}} + {\text{ WF}}_{{{\text{gray}} }}\quad \,\,{\text{m}}^{{3}} {\text{t}}^{{ - {1}}}$$

3 $${\text{WF}}_{{{\text{green}}}} = {\text{CWU}}_{{{\text{green}}}} {\text{Y}}\,\quad{\text{m}}^{{3}} {\text{t}}^{{ - {1}}}$$

4 $${\text{WF}}_{{{\text{green}}}} = {\text{CWU}}_{{{\text{green}}}} {\text{Y}}\,\quad{\text{m}}^{{3}} {\text{t}}^{{ - {1}}}$$

5 $${\text{CWUblue = 10}} \times \sum\nolimits_{{\text{d = 1}}}^{{{\text{lgp}}}} {{\text{ET}}}_{{{\text{blue}}}} \,\quad{\text{m}}^{{3}} {\text{ha}}^{{ - 1}}$$

6 $$CWU{\text{green}} = \,10\, \times \sum\nolimits_{d = 1}^{{{\text{lgp}}}} {{\text{ET}}_{green} } \,\quad{\text{m}}^{3} {\text{ha}}^{ - 1}$$

7 $${\text{ET}}_{{{\text{blue}}}} = {\text{ IR}}\,\quad{\text{mm year}}^{{ - {1}}}$$

8 $${\text{IR }} = {\text{ ET}}_{{\text{C}}} - {\text{Peff}}\,\quad{\text{mm year}}^{{ - {1}}}$$

9 $${\text{ET}}_{{{\text{green}}}} = {\text{ ET}}_{{\text{C}}} - {\text{IR}} \,\quad{\text{mm year}}^{{ - {1}}}$$

10 $${\text{ETc }} = {\text{ Kc }}*{\text{ ET}}_{{\text{O}}} \,\quad{\text{mm year}}^{{ - {1}}}$$

11 $${\text{ETc}} = {\text{ ETc }}*{\text{month in each year}} \,\quad{\text{mm year}}^{{ - {1}}}$$

12 $${\text{WF}}_{{{\text{gray}}}} = \, ((\alpha *{\text{AR}})/\left( {{\text{C}}_{{{\text{max}}}} - {\text{C}}_{{{\text{nat}}}} } \right))/{\text{Y}}\,\quad{\text{m}}^{{3}} {\text{t}}^{{ - {1}}}$$

### Green and blue water evapotranspiration of crops

The water demand of grapevine and olive crops was determined considering the crop water requirement (CWR) using the CROPWAT 8.0 software^73,74^. This software allows the calculation of water supply for different crop patterns, amount of irrigation according to their typology, and crop yield, both under irrigated and non-irrigated conditions^75^.

The crop evapotranspiration (ETc) was calculated based on irrigation requirement (IR), assuming that water losses due to irrigation remain and return to the basin.

Where ETc (mm/dec) is adjusted to decadal values (dec), in relation to irrigation efficiency throughout the crop growing season, under ideal growing conditions, it is assumed that the water requirements of the plantations are met^76^, so that the sum of ET_green_ and ET_blue_ equals ETc.

ET_blue_ was estimated according to Eq. (7), an equation that included the determination of I.R. (Eq. (8). the program calculated Effective Precipitation (Peff) according to the USDA S.C. method. This parameter refers to the fraction of the total precipitation used to meet the irrigation needs of the crop. If (Peff) is higher than the total evapotranspiration of the crop, ET_blue_ is equal to zero indicating that no additional water is needed.

The value of ET_green_ was determined using Eq. (9), which considers the ETc values calculated in Eqs. (10) and (11). The crop coefficient, Kc, considers the crop characteristics and the average effects of soil evaporation. The reference evapotranspiration, ETo (mm month^−1^), was calculated using the Penman–Monteith method with the CROPWAT 8.0 program. Climate data based on the latitude and analyzed period was also considered in the calculation^29^.

### Calculation of the gray water footprint of crops (WF_gray_)

The WF_gray_ is defined as the amount of water necessary to assimilate the residues and dilute them so that their concentration remains within the quality ranges, according to the regulations^38^. WF_gray_ was estimated according to Eq. (12), where AR corresponds to the applied amount of fertilizer, α represents the leaching and runoff fraction of the product expressed as a percentage, C_max_ is the maximum acceptable concentration defined by quality standards, C_nat_ is the natural concentration of the pollutant, Y is the yield of agricultural production, and the agent used corresponds to nitrogen for each agricultural crop (AR) ^72^.

The leaching fraction of this agent was 10%, and the C_max_ was 0.03 kg m^3^, based on NOM-001-SEMARNAT Mexican Emission Standard. The C_nat_ was set at 0.00001 kg m^3^ through the best-case scenario assessment according to the methodology of Hoekstra^72^.

### Statistical analyses

Correlation analyses were performed for the hydrological variables according to the Pearson coefficient using STATISTICA 8.0. In addition, for the WF_agricultural_ ANOVA tests were computed.

## Data Availability

This entire data set, or specific portions of it, supporting the findings of this study are available from the author V.N. on request.

## Abbreviations

AR (kg/hamass/area): Application rate of a chemical (fertilizer or pesticide) per unit of land
α: Leaching-run-off fraction, i.e. fraction of applied Chemicals reaching freshwater bodies
CWU_blue_ (m^3^ ha^−1^/volume/area): Blue crop water use
CWU_green_ (m^3^ ha^−1^/volume/area): Green crop water use
CWR (mm month^−1^/length/time): Crop water requirement
C_max_ (kg m^3^/mass/volume): Maximum acceptable concentration of a chemical in a receiving water body
C_nat_ (kg m^3^/mass/volume): Natural concentration of a chemical in the receiving water body
ET c(mm month^−1^/length/time): Crop evapotranspiration (under optimal conditions)
ET_blue_ (mm year^−1^/ mm month^−1^/length/time): Blue water evapotranspiration
ET_green_ (mm year^-1^/mm month^−1^/length/time): Green water evapotranspiration
ETo mm (month^−1^/length/time): Reference crop evapotranspiration
Igp: Length, days in each stage of the cycle
IR (mm month^−1^/length/time): Irrigation requirement
Kc: Crop coefficient
Peff (mm month^−1^/length/time): Effective rainfall
WF_agricultural_ (m^3^t^−1^/volume/time): Agricultural water footprint
WF_blue_ (m^3^t^−1^/volume/time): Blue water footprint
WF_crops_ (m^3^t^−1^/volume/time): Crops water footprint
WF_gray_ (m^3^t^−1^/volume/time): Gray water footprint
WF_green_ (m^3^t^−1^/volume/time): Green water footprint
WF_grapes_ (m^3^t^−1^/volume/time): Grapes water footprint
WF_olives_ (m^3^t^−1^/volume/time): Olives water footprint
